# Supplementary material for: Emissive brightening in molecular graphene nanoribbons by twilight states
Source: Nat Commun. 2024 Apr 6;15:2985. doi: 10.1038/s41467-024-47139-1 (PMC10998898; doi:10.1038/s41467-024-47139-1)
Supplement: Supplementary file 1 — Supplementary Information [file 41467_2024_47139_MOESM1_ESM.pdf]

Supplementary Information

Emissive Brightening in Molecular Graphene Nanoribbons by Twilight States

Bernd K Sturdza,[†] Fanmiao Kong,[‡] Xuelin Yao,[‡] Wenhui Niu,[§,¶] Ji Ma,[§,¶] Moritz K Riede,[†] Xinliang Feng,[§,¶] Lapo Bogani,[‡,#,] Robin J Nicholas[†,*]*

[†] Department of Physics, University of Oxford, Parks Road, OX1 3PU, Oxford, UK.
[‡] Department of Materials, University of Oxford, 16 Parks Road, OX1 3PH, Oxford, UK.
[§] Center for Advancing Electronics Dresden (CFAED), Faculty of Chemistry and Food Chemistry, Technische Universität Dresden, Mommsenstraße 4, 01062, Dresden, Germany.
[¶] Max Planck Institute of Microstructure Physics, Weinberg 2, 06120, Halle, Germany.
[#] Departments of Chemistry and Physics, University of Florence, V. della Lastruccia, 50019, Sesto Fiorentino, Italy

Corresponding authors: bernd.sturdza@physics.ox.ac.uk, robin.nicholas@physics.ox.ac.uk, lapo.bogani@materials.ox.ac.uk

Supplementary Methods: Graphene nanoribbon sample preparation

Cove-edged graphene nanoribbons (GNRs) with dodecyl side chains (**4-CGNR**) (Supplementary Fig. 1) and cove-edged GNRs decorated with the Diels-Alder cycloadduct of anthracenyl unit and N-n-octadecylmaleimide (**GNR-AOM**) were prepared according to reported procedures [1-2]. Ribbons have a length distribution of L=10-371 nm. AOM side chain as model was prepared following the reported similar synthetic route [3], 0.2 mg/ml GNR-AOM were dissolved in chloroform and briefly sonicated.

For spectroscopic measurements at low temperatures, polystyrene (PS) or ethylene-vinyl acetate (EVA) polymer was added to the GNR solution in a 50:1 polymer to GNR ratio. The solution was consequently deposited on a glass substrate, which was heated to 50 ^{o}C, via drop casting to form a film of GNR in polymer matrix. We used two different transparent polymers, EVA and PS, to investigate possible effects of the polymer matrix. When comparing room temperature spectra of GNR in solution and in polymer matrix we found no effect of the polymer or solvent on PL, Raman or absorbance measurements at concentrations below 0.2 mg/ml in chloroform solution and 1:25 in transparent polymer matrix thin films, see Supplementary Fig. 9.

Supplementary Text 1: Fitting and Franck-Condon modelling the GNR optical response

To fit the optical response, we chose a staged process:

First, we performed a free parameter fit on amplitude, width, and position in order to determine the energy of the transitions and hence the basic mechanism (top of Supplementary Fig. 7). For the PL, this requires eight Lorentzian peaks, one for the ZPL, five for the RBLM-mode, one for the G-mode, and one for low energy excimer emission. For the absorption, we chose nine Lorentzian peaks, one for the ZPL, three for the RBLM-mode, three for the G-mode, one for the E_{12} transition, and one for high energy absorption.

Fitting a single spectrum with this many parameters leaves room for uncertainty in the fitting result as this often leads to multiple solutions with similar accuracy. However, we found that when all parameters are free and independent, the results shown in Supplementary Fig. 7 were robustly reproducible under multiple varying starting parameters. This procedure shows us that the ZPL and 5 RBLM peaks form a single series of peaks with a very accurate spacing equal to the RBLM energy as shown in the inset Fig. 2d.

In the second step, we chose a fit function satisfying a simple Franck-Condon model which imposes conditions on the position, width and amplitude of the phonon peaks.

Franck-Condon modelling was performed according to previous studies [4]. The intensity of a single FC progression coupled to a single phonon mode is calculated as

$$I(\hbar\omega) \propto (\hbar\omega)^3 e^{-S_i} \sum_{\nu_i} \frac{S_i^{\nu_i}}{\nu_i!} \Gamma(\hbar\omega - E_0 + \nu_i \hbar\omega_i)$$

with the intensity I, the photon energy $\hbar\omega$, the Huang-Rhys factor S, the 0-0 transition energy E_0 (E_{ZPL}), phonon energy $\hbar\omega_i$, and the line shape function Γ, which is assumed to be Lorentzian. For our material, the optical response can be modelled with the RBLM and G phonons as well as the zero-phonon line, see Supplementary Fig. 7. Note that the model does not include emission above or absorption below the ZPL energy which is thus excluded.
In addition, the PL data contains a wide Lorentzian contribution from low energy emission due to defects and excimers. Similarly, the absorption has an additional wide Lorentzian contribution at higher energies and a narrow Lorentzian representing the second interband transition E_{12}. Results of the modelling are given in Supplementary Table 2.

For the absorption spectrum, which is dominated by the G-mode, this works relatively well. However, the PL spectrum deviates from the predicted amplitude of a purely Franck-Condon model and we have thus included an extra peak for the 0-1 RBLM phonon to allow the fit function to match the data sufficiently well (Supplementary Fig. 7). Still, the fit function deviates from the data at energies approaching the G-mode peak around 1900meV. The higher RBLM modes at this point are significantly enhanced relative to the simple model which suggests the presence of a second phonon coupling mechanism such as Herzberg-Teller coupling, as discussed in the main text.

Supplementary Text 2: Zone folding of CNT(6,6) and cove-GNR

The electronic band structure of CNT(6,6) can be obtained by superimposing the graphene band structure at allowed k lines, which is the fundamental idea of zone-folding approximation. The first Brillouin zone of graphene is a hexagon, as shown in Supplementary Fig. 10. Assume the C-C bond length in graphene is a, then the coordinates of rightmost K and K' are $\left(2\pi/3a\,, 2\pi/3\sqrt{3}a\right)$ and $\left(2\pi/3a\,, -2\pi/3\sqrt{3}a\right)$ respectively. Suppose the CNT(6,6) and cove-GNR are both periodic along y direction. Since the nanotube is confined in the circumferential direction, its Brillouin zone is a vertical line of length $2\pi/\sqrt{3}a$, as denoted by the green lines in Supplementary Fig. 10. Along the transverse direction there are only discrete allowed wavenumbers. For CNT(6,6) the horizontal k path F-M is divided into six parts. So we projected the band structure of CNT(6,6) on graphene band structure at these discrete k lines, from which we can see the contributions of graphene band structure to each band of CNT(6,6). For example, the highest unoccupied band and lowest occupied band of CNT(6,6) are contributed by the graphene band structure with transverse wavenumber $k_x = 0$. The lowest unoccupied band and highest occupied band are contributed by the graphene band structure with transverse wavenumber $k_x = 2\pi/3a$, i.e. the line passing through K and K' valleys.

In contrast, the transverse wavenumber in cove-GNR is no longer a good quantum number, as the translational invariance in lateral direction is not well defined. Nonetheless we still choose the same k lines to project the band structure of cove-GNR in order to compare with that of CNT(6,6). One important difference of cove-GNR is that the unit cell along periodic direction is three times longer than that of the CNT(6,6). Therefore the first Brillouin zone of cove-GNR is one third of the first Brillouin zone of CNT(6,6). In Supplementary Fig. 11, the k lines are of the same length as Supplementary Fig. 10, but they correspond to three Brillouin zones of cove-GNR now. It can be directly seen from the diagram that both K and K' valleys are folded to Γ point in cove-GNR Brillouin zone. By looking at the projected band structure, we can conclude that the lowest unoccupied bands and highest occupied bands, which are the bands account for the optical transitions, are mainly contributed by the graphene band structure at k path passing through the K and K' points. And the two valleys are completely mixed at the Γ point in cove-GNR Brillouin zone.

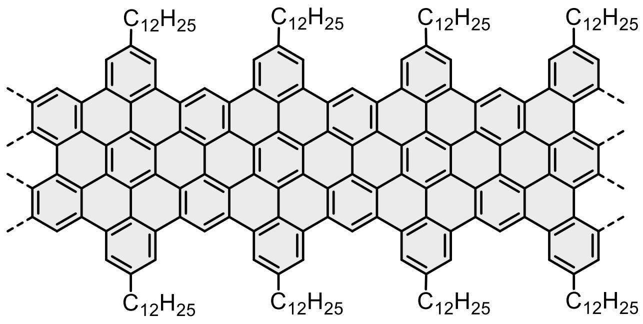

Supplementary Fig. 1. Chemical structure of dodecyl functionalised GNR (4-CGNR).

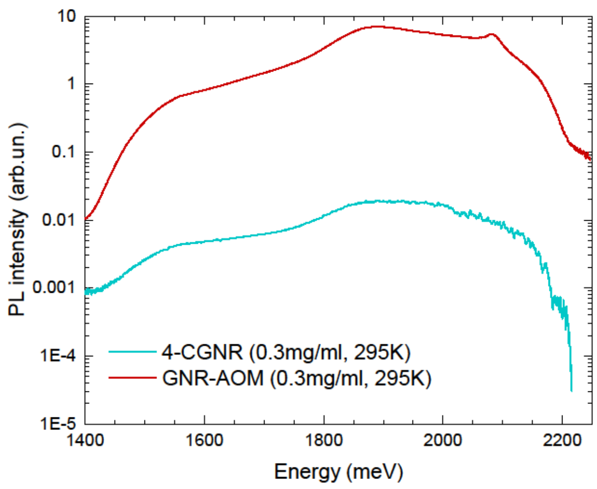

Supplementary Fig. 2. Comparison of PL intensities for GNR with dodecyl side chains (4-CGNR) and with *N*-*n*-octadecylmaleimide (GNR-AOM). Photoluminescence spectra were acquired in chloroform solution with 2.33eV laser excitation. The graphene backbones of the two samples are identical, but the dodecyl side groups are much smaller than the AOM groups and thus do not prevent π- π -stacking of GNRs, which is known to quench the PL response. PL spectra acquired with a laser intensity of $2mW/cm^2$.

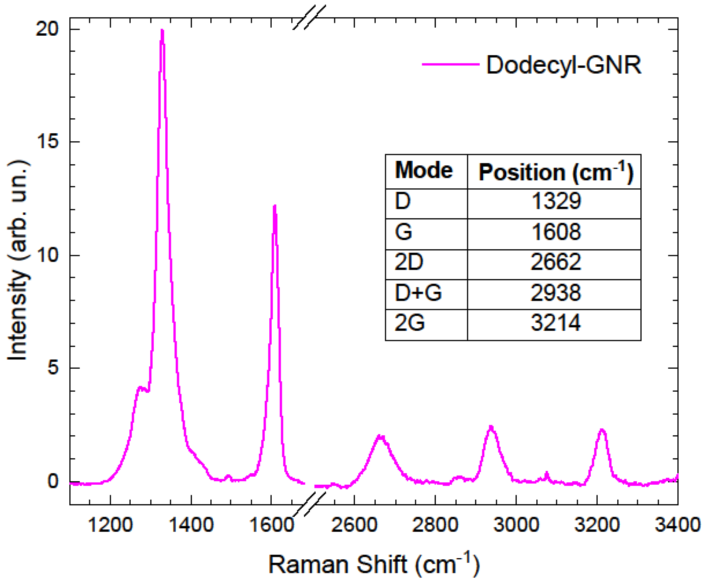

Supplementary Fig. 3. Raman spectrum of dodecyl functionalised GNR (4-CGNR) reference sample. The sample was excited with a 2.33eV laser and displays D, G and their second order Raman modes, positions are noted in the inset. In contrast to the n-octadecylmaleimide functionalised GNR, the second order modes can be clearly resolved here, which is due to the absence of an appreciable PL response.

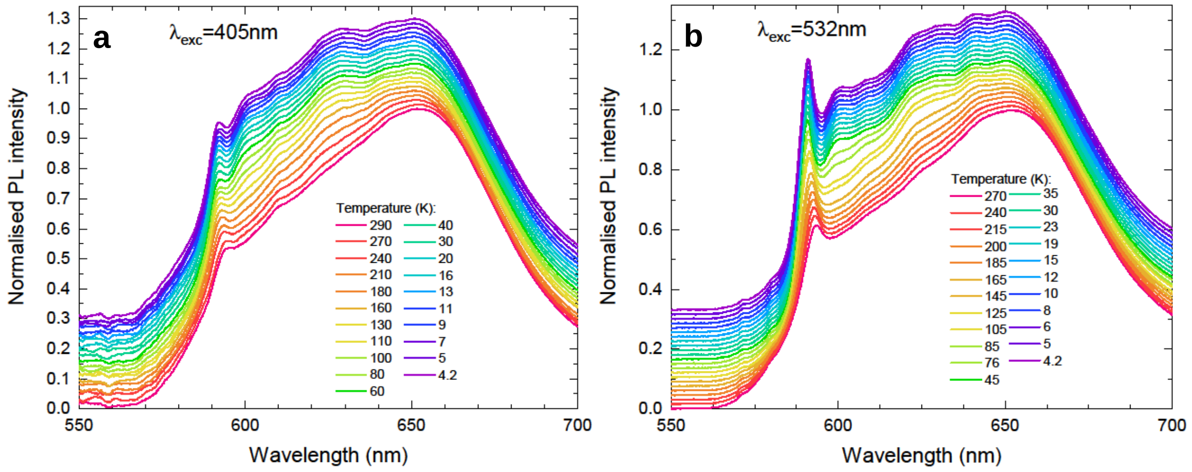

Supplementary Fig. 4. Temperature evolution of PL response of GNR-AOM thin film on a wavelength axis. The sample was excited with **a)** 3.06eV (405nm) and **b)** 2.33eV (532nm) lasers.

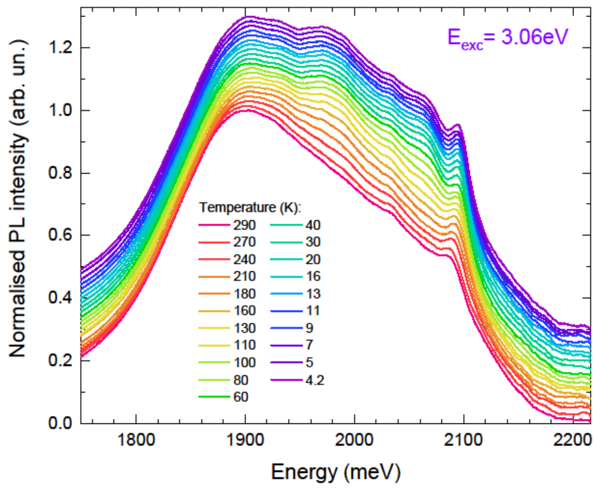

Supplementary Fig. 5. Temperature evolution of PL response of GNR-AOM with 3.06eV excitation. Temperature dependent photoluminescence spectra of GNR-AOM in polymer matrix excited with a 3.06eV laser.

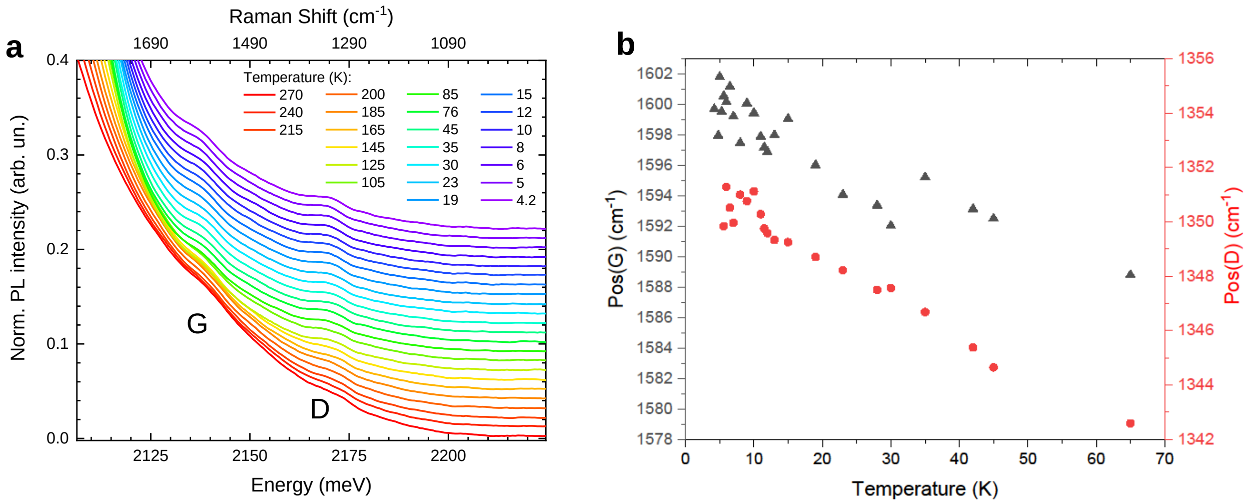

Supplementary Fig. 6. D and G phonon mode positions of GNR-AOM resolved from the low temperature PL of GNR-AOM. a) High energy tail of the PL response of GNR-AOM excited with 2.33eV. At lower temperatures the D and G Raman modes can be resolved in the PL onset. **b)** Low temperature D and G mode positions retrieved via peak fitting. Both modes shift towards lower wavenumbers with increasing temperatures, confirming that phonon scattering rather than thermal expansion drive this shift [5].

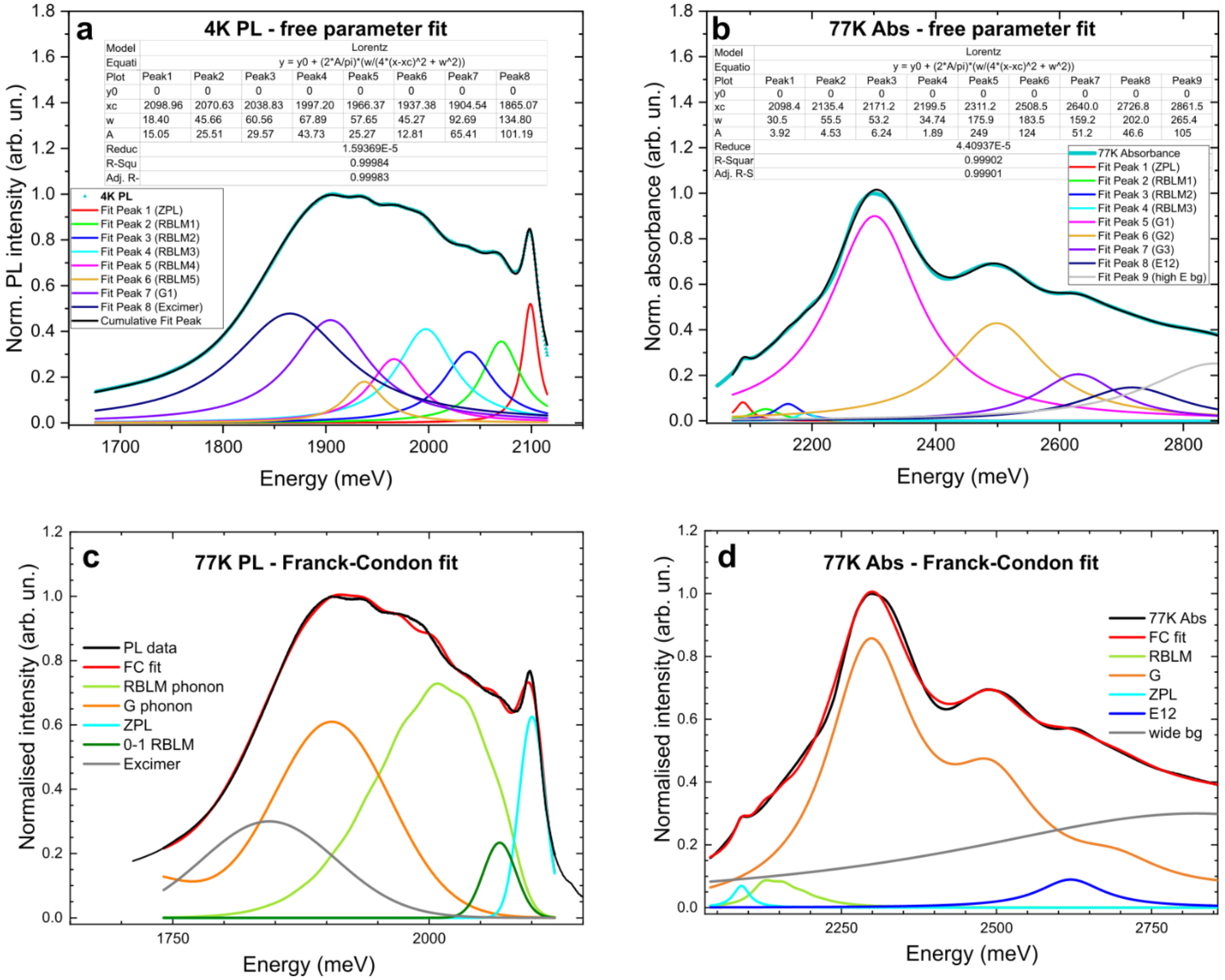

Supplementary Fig. 7. Fitted optical spectra and Franck-Condon modelling of GNR-AOM. a-b) PL and absorption spectra of a GNR-AOM thin film at 4K and 77K with free parameter peak fits. **c-d)** Franck-Condon modelling results of GNR-AOM thin film PL and absorption spectra at 77K. PL data was excited with a 2.33eV laser. The individual contributions as well as the combined spectra are shown. For Franck-Condon model parameters see Supplementary Table 2.

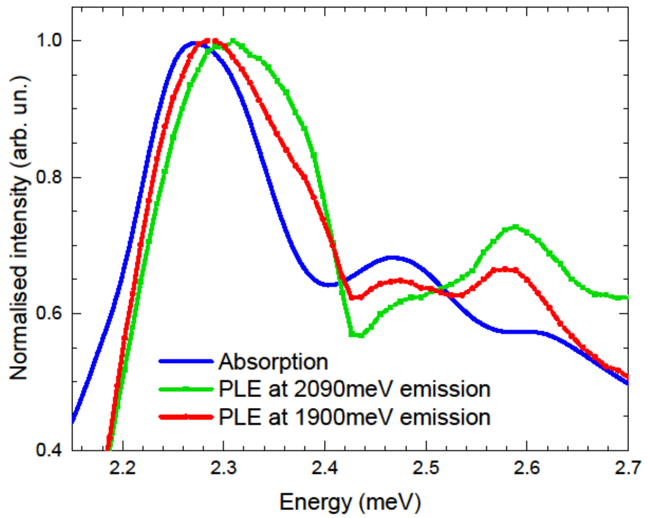

Supplementary Fig. 8. Photoluminescence excitation (PLE) compared to absorption data for GNR-AOM sample in chloroform. The onset around 2.2eV is shifted due to different optical filters used in the two experiments. The peak of the second interband transition E_{21} is more pronounced in the PLE data.

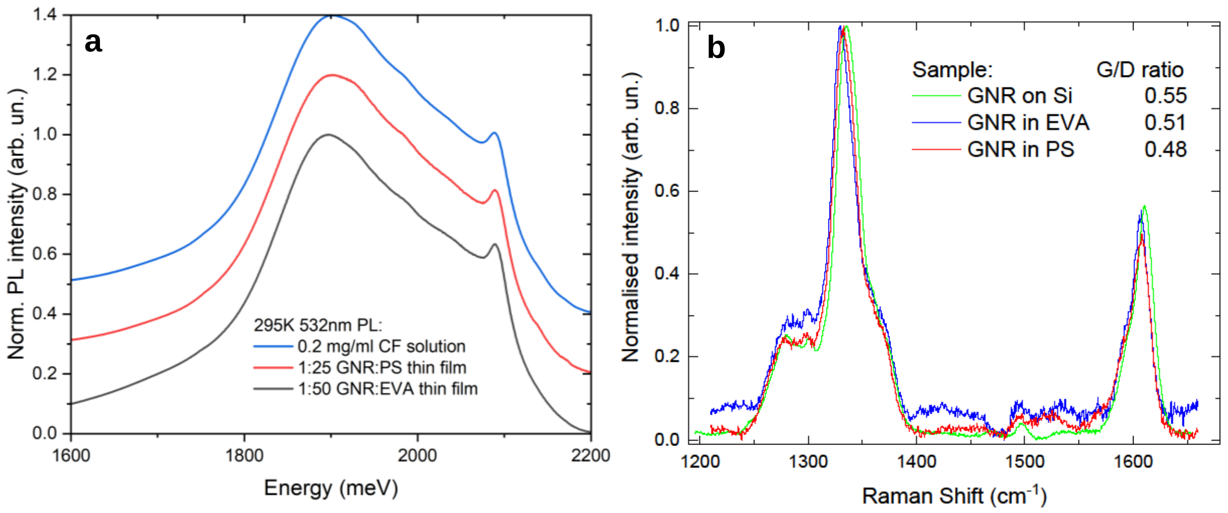

Supplementary Fig. 9. Photoluminescence and Raman spectra of different GNR-AOM samples. a) The PL spectra were taken with 2.33eV excitation at room temperature and normalised. The GNR samples in chloroform solution and in polymer matrix thin films show nearly identical PL spectra. **b)** The Raman spectra of a pure GNR sample deposited on a Silicon wafer and samples at 1:50 ratios in two different transparent polymer matrices show no appreciable differences.

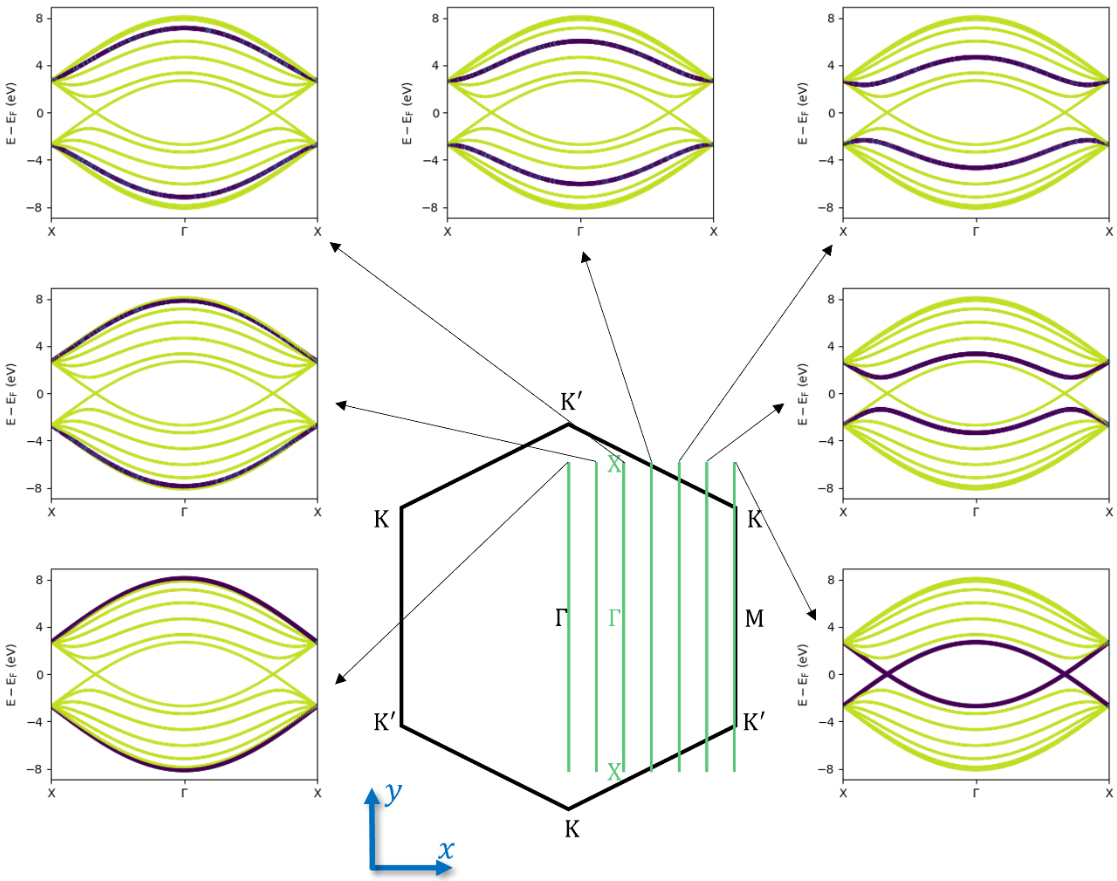

Supplementary Fig. 10. Projected band structures of CNT(6,6) on band structure of graphene at different k path. The green lines are first Brillouin zone of CNT(6,6) with different transverse wavenumbers. The band structure of CNT(6,6) is projected to the band structure of graphene at these discrete k lines. The whole band structure of CNT(6,6) consists of contributions of graphene band structure at all the k lines.

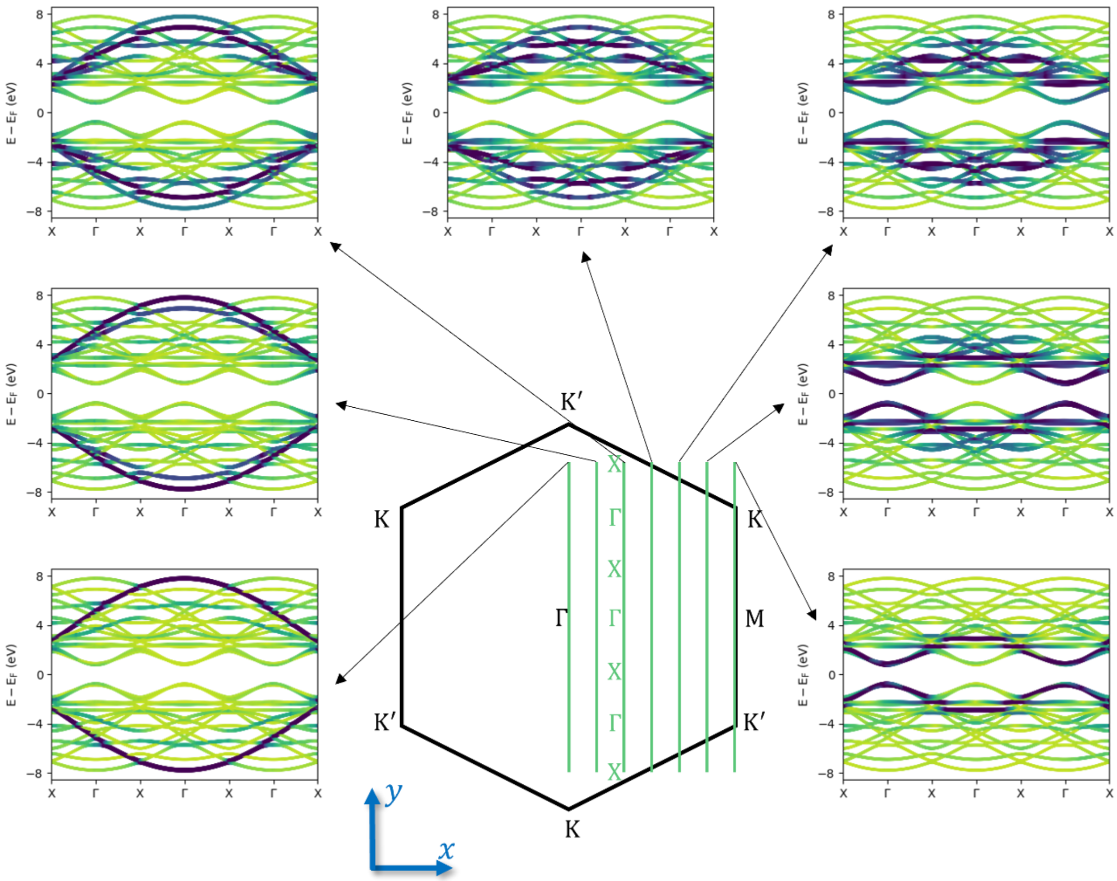

Supplementary Fig. 11. Projected band structures of cove-GNR on band structure of graphene at different k path. The green lines are the same to those in Supplementary Fig 10, except for that the lines here are three extended Brillouin zone of cove-GNR. The band structure of cove-GNR is projected to the band structure of graphene at these discrete k lines. It is not as obvious as CNT(6,6) that how the band structure of graphene is folded to that of cove-GNR.

Supplementary Table 1. Photoluminescence quantum efficiencies (PLQE) of GNR-AOM. Data were acquired in chloroform solution at different concentrations with a 3.06eV laser.

GNR concentration (mg/ml)	Cuvette path length (mm)	OD	PLQE (%)
0.2	1	0.07	6.43
0.066	10	0.26	6.32
0.066	1	0.03	6.25
0.002	10	0.01	6.13

Supplementary Table 2. Fit parameters of Franck-Condon modelling of GNR-AOM at 77K. Results are provided with the precision of the fits for photoluminescence (left) and absorption (right) data.

PL contribution	A	Centre (meV)	S	σ (meV)
ZPL	0.63	X0=2100		13
RBLM	0.32	X0	3.4	17
G	0.26	X0	0.66	57
0-1 RBLM	0.23	X0-31.6		16
Excimer	0.30	1844		62

Abs Contribution	A	Centre (meV)	S	σ (meV)
ZPL	0.07	X0=2099		20
RBLM	0.02	X0	1.42	15
G	0.21	X0	0.57	71
E_{21} peak	0.09	2626		29
High Energy background	0.30	2828		300

Supplementary References

[1] Niu, W., Sopp, S., Lodi, A. et al. Exceptionally clean single-electron transistors from solutions of molecular graphene nanoribbons. Nat. Mater. ***22****, 180–185 (2023).* *https://doi.org/10.1038/s41563-022-01460-6*

[2] Narita, A., Feng, X., Hernandez, Y. et al. Synthesis of structurally well-defined and liquid-phase-processable graphene nanoribbons. Nature Chem ***6****, 126–132 (2014).* *https://doi.org/10.1038/nchem.1819*

[3] Zhao, S., Rondin, L., Delport, G. et al. Fluorescence from graphene nanoribbons of well-defined structure. Carbon ***119****, 235-240 (2017).* *https://doi.org/10.1016/j.carbon.2017.04.043.*
[4] Saidani, M.A., Benfredj, A., Romdhane, S., Kouki, F., Bouchriha, H.: Role of intermolecular coupling and electron-nuclear coupling in the photophysics of oligothiophenes. Phys. Rev. B **86** (16), *1–6 (2012).* *https://doi.org/10.1103/PhysRevB.86.165315*

[5] Liu, H.N., Cong, X., Lin, M.L., Tan, P.H.: The intrinsic temperature-dependent Raman spectra of graphite in the temperature range from 4K to 1000K. Carbon ***152****, 451–458 (2019).* *https://doi.org/10.1016/j.carbon.2019.05.016*
